# Assessing perceptions and priorities for health impacts of climate change within local Michigan health departments

**DOI:** 10.1007/s13412-021-00679-0

**Published:** 2021-05-11

**Authors:** Julie M. Carter, Patricia D. Koman, Lorraine Cameron, Aaron Ferguson, Patrick Jacuzzo, Jason Duvall

**Affiliations:** 1 Program in the Environment, College of Literature, Science, and the Arts and the School for Environment and Sustainability, University of Michigan, 440 Church St, Ann Arbor, MI 48109 USA; 2 Environmental Health Sciences, School of Public Health, University of Michigan, 1415 Washington Heights, Ann Arbor, MI 48109 USA; 3 Division of Environmental Health, Michigan Department of Health and Human Services, Suite 409, PO Box 30037, Lansing, MI 48909 USA; 4 Environmental Health Division, Marquette County Health Department, 184 US 41 East, Negaunee, MI 49866 USA

**Keywords:** Climate, Disaster planning, Greenhouse effect, Health, Health policy, Hospitals, Humans, Local government, Perception, Program development, Public health/methods, Surveys and questionnaires, United States

## Abstract

Climate change affects Michigan’s public health in several primary ways, including increased incidences of vector-borne, waterborne, heat-related, and respiratory illness. Because local health departments (LHDs) play a central role in surveillance and preventative health services, they are among the first institutions to contend with the local impacts of climate change. To assess current perceptions among Michigan public health officials, an online survey was conducted in partnership with the Michigan Association for Local Public Health (MALPH). Most of the Michigan respondents (62%, *n* = 34) agreed that their jurisdictions have experienced climate change in the last 20 years, and 77% agreed that climate change will impact their jurisdictions in the coming 20 years. However, only 35% (*n* = 34) of Michigan officials agreed that climate change is a priority in their departments. About one quarter (25%, *n* = 34) of Michigan LHD respondents did not know about the level of expertise of either the state and federal agencies, responsible for assisting them with information and programs related to climate change and health. Uncertainty regarding the resources available to them may hinder LHDs from developing necessary preparedness, so meeting this need could bolster the public health response to climate change.

**Supplementary Information:**

The online version contains supplementary material available at 10.1007/s13412-021-00679-0.

## Introduction

Over the last several decades, US public health professionals have become increasingly aware of the risks posed by climate change and the need to prepare (Roser-Renouf et al. 2016; McAdams et al. 2019). The health effects associated with climate change include increases in cardiovascular and respiratory morbidity and mortality from air pollution, heat-related illness and death, allergic rhinitis, water-borne infectious diseases (e.g., *Legionella, E. coli*), vector-borne diseases (e.g., West Nile and Lyme disease) (Ogden et al. 2013), injury from severe storms or droughts, and health impacts related to human migration and food insecurity (Ebi et al. 2018; Bell et al. 2018; Balbus et al. 2016; Ogden et al. 2013). Despite an increasing number of multi-billion dollar climate-related disasters in the USA and substantial scientific evidence demonstrating the connection of climate to public health (NOAA National Centers for Environmental Information 2020; Smith and Katz 2013; Watts et al. 2018; Ebi et al. 2018; Watts et al. 2021), the capacity to address climate change by local public health departments is challenged because the US national public health system is chronically under-resourced (Baker et al. 2004; Institute of Medicine 2003; Leider et al. 2020) and local public health has not placed a priority on mitigating or adapting to climate change (Himmelstein and Woolhandler 2016; McCullough et al. 2020). Michigan is no exception, having been left in a state of “public health subsistence” due to low levels of state and federal funding (Citizens Research Council 2018).

At the same time, vulnerable populations have been shown to be adversely affected by climate change-related impacts from environmental and social factors (Watts et al. 2018; Ebi et al. 2018; Frieden 2010). Social determinants of health are generally defined as the conditions where people are born, live, learn, work, play, worship, and age that affect a wide range of health, functioning, and quality-of-life outcomes and risks, including social, built environment, and economic factors. Scholars, public health practitioners, and clinicians are increasingly connecting climate change and social determinants of health (Kresge Foundation 2019; Galvão et al. 2009; Ragavan et al. 2020). Thus, we define climate determinants of health as climate-related conditions present where people are born, live, learn, work, play, worship, and age associated with population health. Climate determinants of health, therefore, recognize and add climate change-related risks to existing socially-patterned vulnerabilities. For example, socially vulnerable populations (e.g., those experiencing poverty or racial discrimination) are at higher risk in heat events for hospitalizations compared to other groups, as they have fewer resources to mitigate the effects of heat and may live in neighborhoods with less heat-mitigating infrastructure (Gronlund 2014; Harlan et al. 2006; Reid et al. 2009). These socially vulnerable populations are also likely to experience multiple environmental stressors (e.g., heat stress, air pollution) compared to other groups in Michigan (Ebi et al. 2018; Koman et al. 2019; Schulz et al. 2016).

Patz et al. (2005) flagged temperate regions, like Michigan, as potentially susceptible to climate change impacts, because they are expected to warm disproportionately and may be less well adapted. The impact of climate change in Michigan is also projected to include extreme heat events, defined as prolonged periods of increased temperatures and humidity, changes in precipitation patterns, including excess rain leading to flooding; and extreme weather such as heavy snow and freezing rain (Ebi et al. 2018; Bidwell et al. 2014; Pryor et al. 2014; Costello et al. 2009). Projected climate-related health burdens in Michigan are estimated at $280 million for extreme heat-associated mortality and $14 million for extreme heat-associated emergency department visits (Gronlund et al. 2019). Thus, a changing climate has serious health implications for present and future generations.

Although local public health authorities are among the primary institutions in the USA to contend with the health impacts of climate change in local communities, little planning has occurred on this scale to explicitly prepare for the climate-related effects (McAdams et al. 2019). In surveys of local public health and planning officials, local governments nationwide (Balbus et al. 2008; Maibach et al. 2008; CDC Foundation 2020) and in Michigan report being underprepared for the consequences of climate change and lacking necessary tools (Norton et al. 2018; White-Newsome et al. 2014). Therefore, understanding how local public health officials are perceiving and preparing for climate-related health risk is essential to climate change adaptation and preparedness efforts (Balbus et al. 2008; Maibach et al. 2008; Watts et al. 2018).

**Fig. 1:**
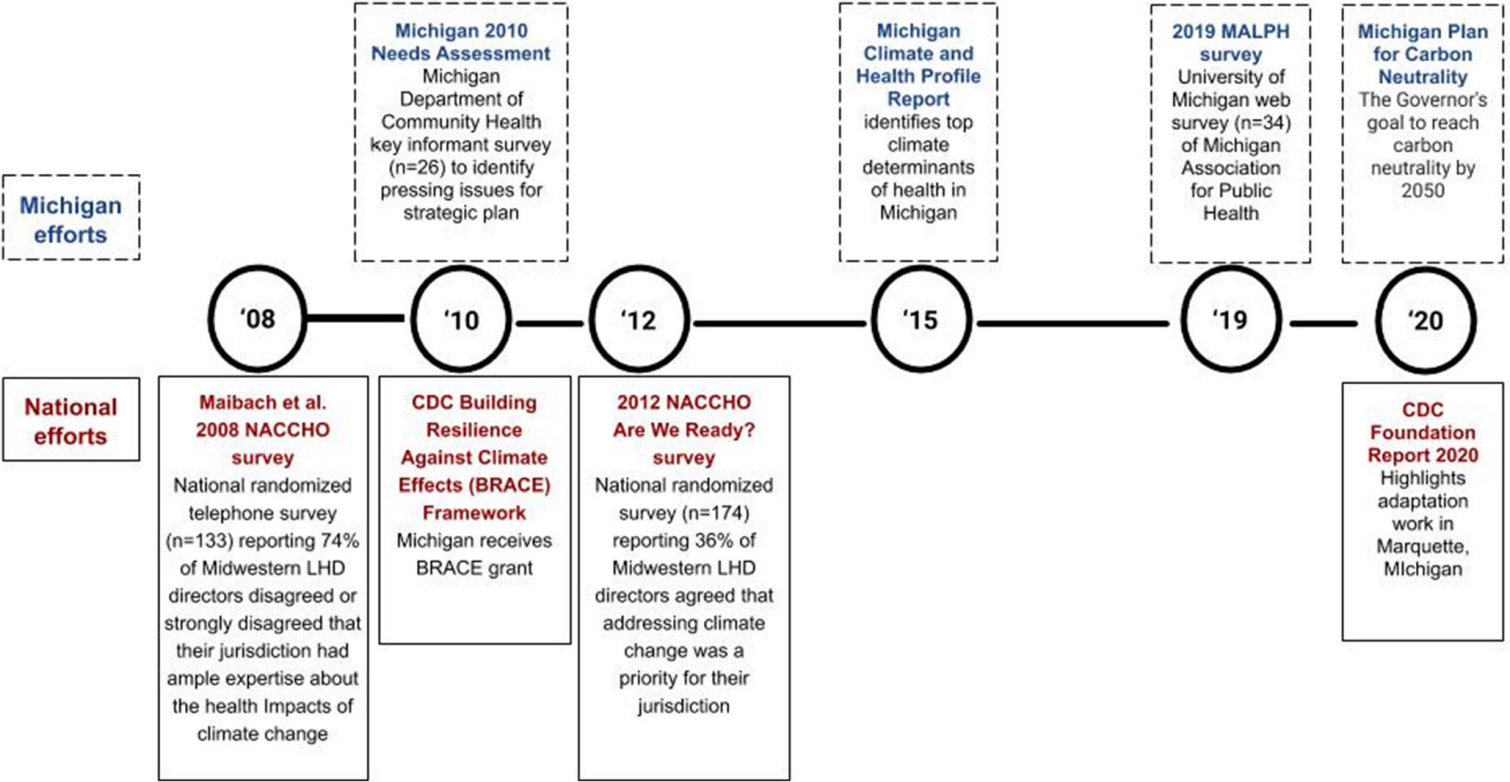
Timeline of notable efforts to understand local health department perceptions of the health impacts of climate change. Activities are at the state level (top): (Michigan Department of Community Health 2010) (Cameron et al. 2015) and at the national level (bottom): (Maibach et al. 2008) (Brown et al. 2012) (CDC Foundation 2020)

A variety of efforts nationally and in the state of Michigan have assessed attitudes of local public health officials regarding climate change and health (Fig. 1). In 2009, with support from the Association of State and Territorial Health Officials (ASTHO), Michigan began planning for climate health adaptation in the state (Michigan Department of Community Health 2010). In a needs assessment, 4% (*n* = 26) of Michigan LHDs reported that climate change was among the top ten current priorities for their jurisdiction (Michigan Department of Community Health 2010).

In the updated 2012 “Are We Ready? Report 2,” a sizeable majority of national LHD directors reported that climate change had affected their jurisdictions in both the 2008 (69%, *n* = 133) and 2012 surveys (66%, *n* = 174) (Brown et al. 2012; Roser-Renouf et al. 2016). Consistent with the 2008 survey results, LHD directors continued to report a lack of the expertise and resources necessary to prepare for and address the health effects. Similar results have been reported more recently in longitudinal surveys of National Environmental Health Association (NEHA) members (McAdams et al. 2019). Midwestern LHD directors reported the lowest self-assessed knowledge and priority of the four regions (Midwestern, Northeastern, Southern, Western) of the USA (Brown et al. 2012). Among Midwestern LHD directors, < 2% strongly agreed, while 20% disagreed, and over half strongly disagreed (54%) that their health department had ample expertise to assess the public health impacts associated with climate change (*n* = 39) (Roser-Renouf et al. 2016).

**Fig. 2:**
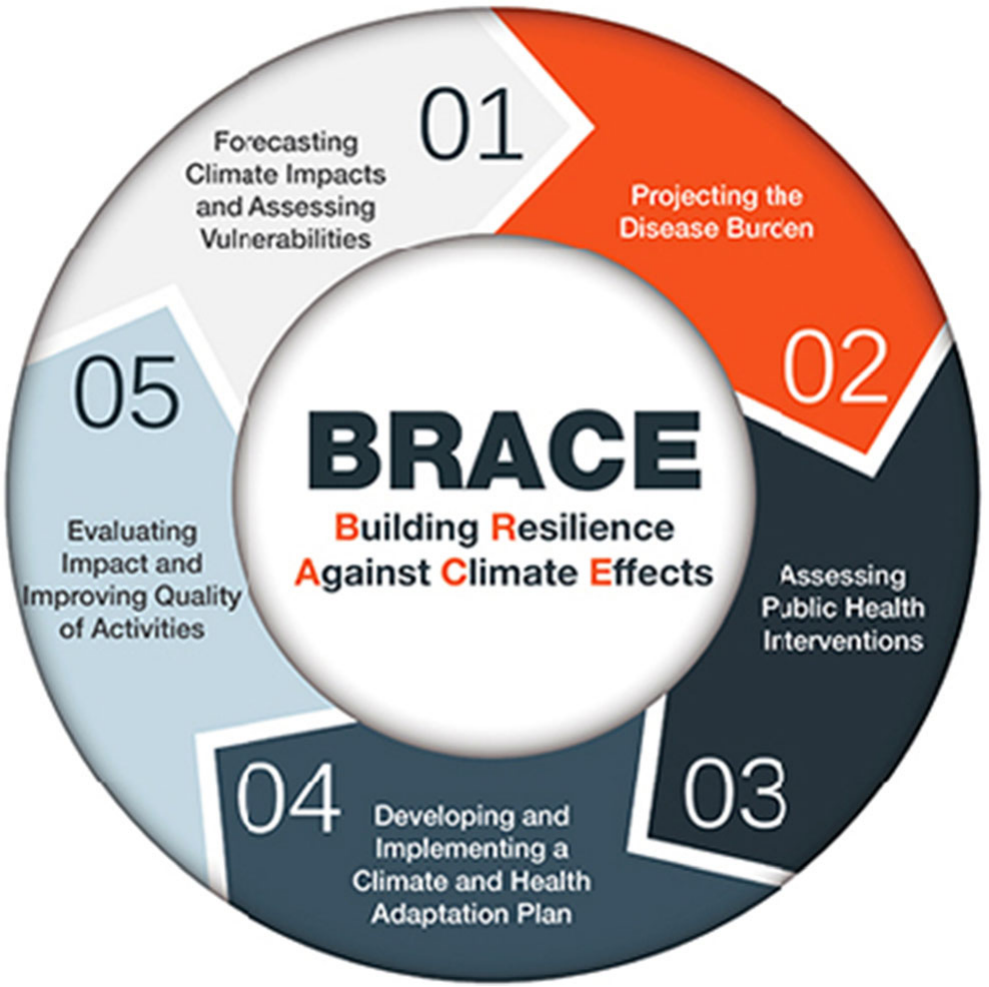
The CDC Building Resilience Against Climate Effects (BRACE) framework is a five-step iterative process that encourages health officials to more effectively anticipate, prepare for, and respond to a range of climate sensitive health impacts (CDC, 2019)

These national surveys raise significant questions regarding the extent to which LHDs have the capacity, expertise, and resources to respond to the impacts of climate change on health, especially in the Midwest. To address the need for preparedness highlighted by these early assessments, in 2010 the U.S. Centers for Disease Control and Prevention (CDC) developed the Building Resiliency Against Climate Effects (BRACE) framework to provide guidance and resources to public health agencies as they develop climate-related health adaptation plans (Marinucci et al. 2014) (Fig. 2).

Over the last 10 years, Michigan has been adopting the BRACE framework, resulting in the Michigan Climate and Health Adaptation Program (MICHAP) strategic plan for 2010–2015 (Cameron et al. 2011), which was updated for 2016–2021 (Cameron and Ferguson 2016). Several Michigan cities have also published climate action plans which considered health adaptation, including Ann Arbor, Detroit, Grand Rapids, Marquette, and Traverse City (City of Ann Arbor Climate Action Plan 2012; Detroit Climate Action Plan 2017; Occhipinti and Ferguson 2013; City of Marquette 2013; Townsend et al. 2011; Woodruff and Stults 2016).

Building from this foundation, researchers at the University of Michigan partnered with the Michigan Association for Local Public Health (MALPH) to survey Michigan LHD officials to answer three primary research questions and to situate Michigan attitudes among those reported in national surveys (Maibach et al. 2008; Roser-Renouf et al. 2016):

1. How are Michigan LHD officials perceiving the risks to public health posed by climate change?
2. How are Michigan LHD officials perceiving key stakeholders’ level of knowledge and expertise in managing the health impacts of climate change?
3. How are Michigan LHD officials perceiving the value of state and CDC frameworks to address the health impacts of climate change?

## Methods

To understand the perceptions of Michigan’s LHD officials in 2019, researchers at the University of Michigan partnered with the Michigan Association for Local Public Health (MALPH) to conduct a survey on climate change and health. We based the survey instrument on the Maibach et al. (2008) published survey instrument with four additional questions designed to address the current study’s third research question. The State of Michigan is comprised of 83 counties and is served by 45 LHDs. 32 LHDs have jurisdiction over a single county, and 12 district-level LHDs serve multiple counties. An additional LHD serves the city of Detroit.

### Sample population and survey distribution

The sample population for this survey consisted of public health officials at the 45 LHDs in Michigan. A survey investigating perceptions of climate change was distributed in partnership with MALPH. MALPH is a professional association that educates and advocates for local public health jurisdictions (www.malph.org). In this role, MALPH is a credible source for communication and their involvement was desired to increase the response rate to the survey. MALPH maintains an up-to-date email directory of LHD officials serving in various professional capacities. We used this directory to distribute an electronic survey link and invitation message explaining the study’s purpose to a total of 44 Health Officers and 81 Environmental Health Directors and senior staff from all 45 LHDs in the state; a vacancy at the time of distribution resulted in the survey being delivered to 44, rather than 45, Health Officers. The initial respondent pool included officials in these positions due to their leadership roles, as well as their potential knowledge of department programs related to climate change. Approximately two months after an initial request for survey responses was sent out through MALPH’s email lists, a second reminder request for responses was sent out in the same manner. The email invitation accompanying both requests asked recipients to forward the survey to additional colleagues to encourage snowball sampling. There was no limitation on type of recipient.

### Survey instrument and measures

An electronic survey was developed using Qualtrics, an online survey platform. The 2019 survey instrument consisted of eleven multiple-choice and rating scale questions, as well as three short fill-in questions concerning background and interview contact information (see Supplementary Materials). We modeled the majority of the questions after those explored in Maibach et al. 2008, and we added four questions regarding familiarity with and value of specific state or federal adaptation plans.

The 2019 Michigan survey included questions to identify each respondent’s position, the county or district served by their health department, and length of employment within that department and evaluated perceptions of climate change impacts and the level of priority. We evaluated eight health issues the survey, including five climate-related health outcomes the Michigan Department of Health and Human Services (MDHHS) identified as priorities for Michigan: respiratory diseases, heat-related illnesses, waterborne diseases, vector-borne diseases, and injury resulting from extreme weather events (Cameron et al. 2015). Seven of these health impacts were identical or very similar to health issues included by Maibach et al. 2008, with the one exception being “injury from extreme weather events.”

We explored perceptions of personal and departmental knowledge and the knowledge of key external groups about the health impacts of climate change using a five-point Likert-type scale (*none* to *a lot*), with an additional “don’t know” option. While the wording of these questions was slightly revised to increase readability, each of the items were similar to those included in the Maibach et al. 2008 survey instrument. 2019 Michigan survey respondents were asked to rate how familiar they and their departments were with climate change adaptation frameworks developed by both the MDHHS-MICHAP strategic plan and the CDC. Familiarity with and value of specific state or federal adaptation plans were not investigated by Maibach et al. 2008 or Roser-Renouf et al. 2016.

### Data analysis

Data from the survey was analyzed using IBM SPSS Version 25 and Microsoft Excel 2016. We generated descriptive statistics to determine frequencies, percentages, and median scores on all rating scale items. To calculate median values, survey responses were coded as numerical values with higher agreement, familiarity, commonness, or value as higher numeric scores. For example, *Strongly agree* was coded as 4, *Agree* as 3, *Disagree* as 2, and *Strongly Disagree* as 1. “Don’t know” answers were treated as missing. As a sensitivity analysis (not shown), “Don’t know” responses were coded as a neutral value, but this treatment would have skewed the median because of the high frequency in the data. For example, the percentage of “Don’t know” answers ranged from 24 to 76% regarding perceptions about current and future climate change impacts.

## Results

### Survey respondents

Twenty-seven of the 45 LHDs (60%) responded representing both urban and rural parts of Michigan; some LHDs had more than one responder. In total, 34 public health officials from 18 county departments and 8 district departments, as well the Detroit Health Department, completed the 2019 online survey. The majority of survey respondents (85%, *n* = 34) identified their position as Environmental Health Director, Administrator, Division Director, Supervisor, or Manager (Table 1). About 15% identified as Health Officer or Medical Director. Given their more senior positions, respondents tended to have significant public health experience, with 53% indicating they had worked in their current LHD for more than 15 years.

**Table 1:** Respondent position and length of professional experience (*n* = 34)

Position^b^	Percent
Environmental Health Director /Administrator	41
Environmental Health Supervisor/Supervisor/ Coordinator or Emergency Preparedness Coordinator	24
Environmental Health Manager /Division Director/Division Supervisor	20
Health Officer or Medical Director	15
Years of experience	
1 to < 5 years	26
5 to 15 years^b^	21
More than 15 years	53

^b^We combined responses for categories with more than 1 and less than 5 responses

### Survey results

The survey results are presented in order of this study’s three main research questions.

#### Research Question 1: How are Michigan local health officials perceiving the threats to public health posed by climate change?

A majority (62%, *n* = 34) of survey respondents agreed (or strongly agreed) that their jurisdictions have experienced climate change in the last 20 years, and a larger majority (76%) agreed (or strongly agreed) that their jurisdictions will experience climate change in the next two decades (Table 2). Over a quarter of the survey respondents (26%) indicated that they did not know if their jurisdictions had already experienced climate change. When asked about the impact of climate change in the future, only 15% of respondents expressed this uncertainty. Despite majorities of respondents agreeing that their jurisdictions have or will experience climate change, 56% disagreed (or strongly disagreed) that preparing for the public health effects of climate change was a priority for their health departments. Slightly more than one third (35%) of respondents agreed that dealing with the health impacts of climate change was a priority for their health departments. None of the respondents strongly agreed with this position.

**Table 2:** Michigan LHD responses about the impact and prioritization of climate change (*n* = 34)

	Percent
My jurisdiction has experienced climate change in the past 20 years. (Median: 3, Agree)^a^	
Strongly Agree	15
Agree	47
Disagree or Strongly Disagree^b^	12
Don’t Know	26
My jurisdiction will experience climate change in the next 20 years. (Median: 3, Agree)^a^	
Strongly Agree	29
Agree	47
Disagree or Strongly Disagree^b^	9
Don’t Know	15
Preparing to deal with the public health effects of climate change is a priority of my health department. (Median: 2, Disagree)^a^	
Strongly Agree	0
Agree	35
Disagree or Strongly Disagree^b^	56
Don’t Know	9

^a^Median values were calculated by numerically coding the answers as *Strongly Agree* = 4, *Agree* = 3, *Disagree* = 2, *Strongly Disagree* = 1, *Don’t Know* coded as missing

^b^We report combined responses for categories with more than 1 and less than 5 responses

When asked about a range of specific health outcomes associated with climate change, excluding those who answered “*don’t know*,” at least half of respondents agreed that the following climate-related health impact have already affected their jurisdiction: heat waves and heat-related illness, vector-borne disease, air quality and respiratory diseases, and access to health care services for people with chronic conditions (Table 3). For each of the eight identified health issues, at least a quarter of respondents indicated for each category that they did not know whether climate change had already affected that issue (ranging from 26 to 76% answering *don’t know*).

**Table 3:** Michigan LHD perceptions of current climate change impacts (*n* = 34)

Question text: “Has climate change already affected the following health issues in your jurisdiction?”	Percent			Median^a^
	Yes	No	Don’t Know	
Heat waves and heat-related illness	38	26	35	2, Yes
Waterborne disease	24	38	38	1, No
Vector-borne disease	53	21	26	2, Yes
Air quality and respiratory diseases	26	21	53	2, Yes
Unsafe/ineffective sewage and septic operation	15	53	32	1, No
Healthcare services for people with chronic conditions during service disruptions, such as extreme weather events	32	21	47	2, Yes
Anxiety, depression, or other mental health conditions	12	12	76	1.5^b^
Injury from extreme weather events	21	26	53	1, No

^a^Median values were calculated by numerically coding the answers as *Yes* = 2, *No* = 1, *Don’t Know* coded as missing

^b^A median value of 1.5 indicates an even split between responses

At least one fifth (range from 21 to 66%) of respondents (*n* = 34) agreed that each of eight identified health impacts will be made more common or severe by climate change in their jurisdictions over the next 20 years (Table 4). At least a quarter of respondents indicated that they did not know whether climate change will impact each of the eight health issues presented (range from 24 to 59%).

**Table 4:** Michigan LHD perceptions of future climate change impacts (*n* = 34)

Question text: “Over the next 20 years, will climate change make this issue *more* common or severe, *less* common or severe, or will it remain the *same* in your jurisdiction?”	Percent			
	More	Same or Less	Don’t Know	Median^b^
Heat waves and heat-related illness	56	12	32	3, More
Waterborne disease	35	30	35	3, More
Vector-borne disease	66	9	25	3, More
Air quality and respiratory diseases	50	12	38	3, More
Unsafe/ineffective sewage and septic operation (*n* = 33)^a^	27	48	24	2, Same
Healthcare services for people with chronic conditions during service disruptions, such as extreme weather events	44	12	44	3, More
Anxiety, depression, or other mental health conditions	21	21	59	2.5^c^
Injury from extreme weather events	38	18	44	3, More

^a^34 respondents answered this question, but one respondent left an element blank; thus, 33 answered the Unsafe/ineffective sewage septic operation item

^b^Median values were calculated by numerically coding the answers as *More* = 3, *Same* = 2, *Less* = 1, *Don’t Know* coded as missing

^c^A median value of 2.5 indicates an even split between responses 2, *Same* and 3, *More*

#### Research Question 2: How are Michigan local health officials perceiving other stakeholders’ level of knowledge and expertise in managing the health impacts of climate change?

While 47% of respondents indicated that they personally know either *a lot* or *a good deal* about the impacts of climate change on health (Table 5), they perceived other stakeholders in their jurisdictions to have significantly less knowledge regarding this topic. For example, 36% answered that senior-level managers in their LHDs know either *a lot* or *a good deal*, and 15% answered that relevant appointed officials have similar levels of knowledge on the health impacts of climate change. Few of the respondents indicated that either elected officials, business owners, or leaders of the healthcare delivery system in their jurisdictions to have *a lot* or *a good deal* of knowledge in this area. The perception of appointed officials’ knowledge (50% were perceived to have at least *some* knowledge) was higher than elected officials (15% were perceived to have at least *some* knowledge), business owners (12% were perceived to have at least *some* knowledge), or leaders of the healthcare system (29% were perceived to have at least *some* knowledge). The median value for elected officials and business owners was *a little* knowledge, and other groups were perceived to have a median value of *some* knowledge.

**Table 5:** Perceived levels of local health department and stakeholder knowledge of health impacts of climate change (*n* = 34)

Question text: “How much do the following stakeholders in your county/district know about the health impacts of climate change?”	Percent						Median^b^
	A lot^a^	A good deal	Some	A little	None	Don’t know	
Local Health Department Perceptions							
You personally	47		47	6		0	3, Some
Senior managers in your department	36		44	9		12	3, Some
LHD View of External Stakeholders’ Perceptions							
	A lot	A good deal	Some	A little	None	Don’t know	Median^b^
Relevant appointed officials^c^	0	15	35	18		32	3, Some
Elected officials	15			38	21	26	2, A little
Business owners	0	12		29	21	38	2, A little
Leaders of the healthcare delivery system including hospitals and medical groups	29^d^			24		47	3, Some

^a^We report combined responses for categories with more than 1 and less than 5 responses

^b^Median values were calculated by numerically coding the answers as *A lot* = 5, *A good deal* = 4, *Some* = 3, *A little* = 2, *None* = 1, *Don’t Know* coded as missing

^c^Appointed officials such as environmental, agricultural, wildlife, energy, and transportation officials

^d^The value for *A good deal* was zero

Another key repository of knowledge of the health effects of climate change resides in adaptation plans. In addition to exploring general stakeholder knowledge, the survey included several questions designed to assess the expertise of local, state, and federal agencies in creating climate adaptation plans focused on public health. About 18% (*n* = 34) of respondents reported that their health department has *a lot* or *a good deal* of expertise in creating public health-oriented climate change adaptation plans, with an additional 29% answering that their departments have *some* expertise (Table 6).

**Table 6:** Perceived levels of organizational expertise in creating effective, health-promoting climate change adaptation plans (*n* = 34)

Question text: “How much expertise do the following groups have in creating effective climate change adaptation plans focused on public health?”	Percent						Median^b^
	A lot^a^	A good deal	Some	A little	None	Don’t know	
Your health department	18		29	29	18	6	2.5^c^
Michigan Department of Health and Human Services (MDHHS)	0	32	26	12		29	3, Some
The Centers for Disease Control (CDC)	24	29	21		0	26	4, A good deal

^a^We report combined responses for categories with more than 1 and less than 5 responses

^b^Median values were calculated by numerically coding the answers as *A lot* = 5, *A good deal* = 4, *Some* = 3, *A little* = 2, *None* = 1, *Don’t Know* coded as missing

^c^The median is between 2, *A little* and 3, *Some*

More respondents perceived the MDHHS (32%, *n* = 34) as having *a good deal* and the CDC (53%, *n* = 34) as having *a lot* or a *good deal* of expertise in creating effective adaptation plans. The median value for the local level was between *a little* and *some* knowledge, at state level was *some* knowledge and at the federal level was *a good deal* of knowledge about adaptation plans focused on health. Over a quarter of respondents reported they did not know about the expertise of the state (29%) and CDC (26%).

#### Research Question 3: How are Michigan local health officials perceiving the value of state and national frameworks to address the health impacts of climate change?

To explore perceptions of current adaptation frameworks, respondents were asked about their familiarity with, and the value of, state and national-level adaptation frameworks. Of the overall survey respondents, relatively few answered these questions at the end of the survey about external stakeholders’ level of familiarity.

First, respondents assessed familiarity with the state strategic plan at their LHDs and among a limited set of external stakeholders (Cameron et al. 2011; Cameron and Ferguson 2016). None of the respondents indicated that any of the identified parties were *extremely familiar* with the MDHHS-MICHAP’s strategic plan (Table 7). Thirty percent indicated that they personally were at least *somewhat familiar* with the plan, with an additional 30% indicating that they were *slightly familiar* with it. 31% of respondents perceived that their LHDs overall were at least *somewhat familiar* with the MICHAP strategic plan, and 35% reported that their LHDs are *slightly familiar* with it. About 59% of respondents (*n* = 14) indicated that appointed officials in their jurisdictions have any degree of familiarity with the state’s strategic plan. A much smaller proportion of respondents (29%, *n* =1 2) indicated that elected officials in their jurisdictions have any degree of familiarity with the state’s strategic plan.

**Table 7:** Perceived levels of familiarity with the Michigan Climate and Health Adaptation Program (MICHAP) strategic plan

Question text: How familiar are the following individuals/groups with the MICHAP strategic plan?	Percent^a^						Median^b^
	Extremely	Very	Somewhat	Slightly	Not at all	Don’t know	
Local Health Department Perceptions							
Yourself (*n* = 30)	0	30		30	40	0	2, Slightly
My health department (*n* = 23)	0	31		35	35	0	2, Slightly
LHD View of External Stakeholders’ Perceptions							
Relevant appointed officials^c^ (*n* = 12)	0	42		17	42	0	2, Slightly
Relevant elected officials (*n* = 14)	0	0	29		71	0	1, Not at all familiar

^a^We report combined responses for categories with more than 1 and less than 5 responses

^b^Median values were calculated by numerically coding the answers as *Extremely familiar* = 5, *Very familiar* = 4, *Somewhat familiar* = 3, *Slightly familiar* = 2, *Not at all familiar* = 1, *Don’t Know* coded as missing

^c^Appointed officials such as environmental, agricultural, wildlife, energy, and transportation officials

Additionally, the value of the state strategic plan to the respondent’s department was assessed. Given the lack of familiarity with the plan, it is not surprising that relatively fewer respondents answered this question (*n* = 14), and many but not all of respondents who previously expressed they were not at all familiar with the plan did not respond to this question. When asked about the value of the MICHAP strategic plan, no respondents found it *extremely valuable* and 36% of respondents indicated that the framework had been *very valuable* or a *somewhat valuable* resource to their LHDs (Table 8). An additional 36% indicated that the plan had been *slightly valuable*, and 29% indicated that the plan had not been at all valuable.

**Table 8:** Michigan LHD perceived departmental value of the MICHAP strategic plan (*n* = 14)

Question text: To what degree has the MICHAP strategic plan been a valuable resource to your department?	Percent					Median
	Extremely	Very or Somewhat	Slightly	Not at all	Don’t know	
My health department (*n* = 14)	0	36	36	29	0	2, Slightly valuable

With respect to the CDC’s BRACE framework, 24% (*n* = 33) of respondents indicated that they, individually, were at least *somewhat familiar,* although no respondents reported being very familiar with the BRACE framework (Table 9). An additional 15% responded that they were *slightly familiar* with the framework, with 60% responding they were not at all familiar or didn’t know. Regarding their health department as a whole, no respondents agreed the department was extremely familiar, 15% of respondents answered that their health department was at least *somewhat familiar* with BRACE, with an additional 24% indicating that their department was *slightly familiar* the framework. About 39% of respondents thought their health department was *not at all familiar* with BRACE, and 21% didn’t know.

**Table 9:** Michigan LHD perceived levels of familiarity with CDC’s BRACE framework

Question text: How familiar are the following individuals/groups with the CDC’s BRACE framework?	Percent^a^						Median^b^
	Extremely	Very	Some-what	Slightly	Not at all	Don’t Know	
Local Health Department Perceptions							
Yourself (*n* = 33)	24^c^			15	57	--^f^	1, Not at all
My health department (*n* = 33)	0	15		24	39	21	1.5^d^
LHD View of External Stakeholders’ Perceptions							
Relevant appointed officials^e^ (*n* = 33)	0	0	18		18	64	1.5^d^
Relevant elected officials (*n* = 32)	0	0	0	44		56	1, Not at all
Michigan Department of Health and Human Services (*n* = 33)	0	21		15		64	3, Some-what familiar

^a^We report combined responses for categories with more than 1 and less than 5 responses

^b^Median values were calculated by numerically coding the answers as *Extremely familiar* = 5, *Very familiar* = 4, *Somewhat familiar* = 3, *Slightly familiar* = 2, *Not at all familiar* = 1, *Don’t Know* coded as missing

^c^Zero respondents reported being very familiar with the BRACE framework

^d^The median is between 2, *Slightly familiar* and 1, *Not at all familiar*

^e^Appointed officials such as environmental, agricultural, wildlife, energy, and transportation officials

^f^Fewer than 5 respondents answered “don’t know” but this category could not be meaningfully combined with another

No respondents perceived external stakeholders in Michigan to be extremely familiar with the BRACE framework. Although the state of Michigan is a BRACE grant recipient, more than half of respondents indicated that they did not know how familiar appointed officials, elected officials, and the MDHHS may be with the CDC BRACE framework. The reported median value for the level of familiarity of the MDHHS staff was higher than other stakeholder’s perceptions. About 21% (*n* = 33) of respondents answered that the MDHHS was at least *somewhat familiar* with the CDC’s BRACE framework, and 18% (*n* = 33) indicated that appointed officials were at all familiar with the framework. Few respondents indicated that elected officials had any familiarity with the BRACE framework.

Only about a third of the respondents chose to answer the question about the value of the CDC’s BRACE framework to their health department (*n* = 11). Among those responding, 55% of respondents answered that the approach was at least *slightly valuable* to their health departments. About 45% indicated that the framework had been of no value at all (Table 10).

**Table 10:** Perceived local health department value of CDC’s BRACE framework (*n* = 11)

Question text: To what degree has the BRACE framework been a valuable resource to your department?	Percent^a^						Median^b^
	Extremely valuable	Very valuable	Some-what valuable	Slightly valuable	Not at all valuable	Don’t know	
My health department	0	55			45	0	2, Slightly valuable

^a^We report combined responses for categories with more than 1 and less than 5 responses

^b^Median values were calculated by numerically coding the answers as *Extremely valuable* = 5, *Very valuable* = 4, *Somewhat valuable* = 3, *Slightly valuable* = 2, *Not at all valuable* = 1, *Don’t Know* coded as missing

## Discussion

Understanding how US LHD officials are perceiving, preparing for, and developing capacity to address climate-related health risk is foundational to the country’s ability to adapt for the future. Climate change is an amplifier of public health threats (Costello et al. 2009). Therefore, climate change is expected to create health needs that exceed current local capacity to respond. It is unknown if simply more resources are needed to support existing public health approaches or if novel, transformational approaches will be necessary (Hess et al. 2012). Our study pointed to major knowledge gaps about some of the basics of the relationship between climate change and health among Michigan LHDs and other key stakeholders, as indicated by both the high numbers of “don't know” answers, and the low median scores among respondents. Furthermore, the survey respondents also reported extremely low levels of familiarity with state and national planning efforts or did not reply, as well as a corresponding lack of perceived value of the state strategic plan and BRACE framework.

### Research Question 1: Perceptions of the risks to health posed by climate change

Despite general agreement with the importance of climate change to health, this survey identified significant gaps in understanding of climate-related health effects among Michigan LHD officials. A central challenge for public health officials is the disconnect between the evidence for health impacts (Ebi et al. 2018; Ebi et al. 2018) and marshalling resources and public support to prioritize adaptation (Hess et al. 2014; Marans et al. 2018). Local health departments’ priorities may be influenced by the views and priorities of their local community and elected officials. Michigan LHD respondents were more likely than the general public to agree that climate change is already affecting public health. In a 2019 Yale University Program on Climate Change Communication opinion poll, 46% of the general public in Michigan responded that climate change is already harming people in the USA and 55% of this group responded that climate change would harm people in the USA in the future (Howe et al. 2015).

Large proportions of Michigan LHD respondents indicated that they did not know how climate change had (range 26–76%, *n* = 34) or would (range 25–59%, *n* = 34) influence priority health issues (Tables 3 and 4) (Cameron et al. 2013, 2015). This lack of knowledge was particularly high for the categories of mental health, air quality-related respiratory disease, and injury or lack of health care access from extreme weather events. This is significant because in the USA, the lack of planning for adequate mental health and health care services during and in the aftermath of major storms has resulted in long-term impacts, particularly for vulnerable populations (Dodgen et al. 2016; Runkle et al. 2018). These areas also require intersectional partnerships with other agencies and the healthcare system, but the substantial amount of *don’t know* responses indicate that LHD respondents may be having difficulties communicating with their potential partners in preparing for climate change in Michigan.

Although respondents were mainly experienced environmental health managers or directors, the low median responses consistently demonstrate a lack of knowledge in this area among many LHDs, rather than the full fluency across the state required for an effective public health response. For example, the self-reported familiarity with the MICHAP strategic plan was 2 (*slightly familiar*), with 40% reporting no familiarity at all. This self-reported lack of knowledge results in Michigan LHDs being unable to track, plan for, request resources, mitigate greenhouse gas emission sources, and respond to health threats from climate change already occurring in the state. Low levels of knowledge also prevent LHDs from playing leadership roles in their communities by helping key groups understand and ameliorate climate-related health impacts. These gaps point to opportunities for education and partnership to bolster public health preparedness, especially as the public increasingly trusts health professionals as a source of climate information (Speiser and Hill 2021).

### Research Question 2: LHD perceptions of key Michigan stakeholders’ knowledge about health impacts of climate change

The survey revealed most respondents perceived other stakeholders in their jurisdictions (e.g., elected officials and business and health system leaders) to have substantially less knowledge of this topic than LHD respondents themselves and their senior managers. LHD respondents rated local officials such as environmental, agricultural, wildlife, energy, and transportation officials, as *not at all* or only *slightly* familiar with state strategic plans and the federal BRACE framework.

Approximately one quarter to almost half (26–47%, *n* = 34) of LHD respondents did not know the level of knowledge regarding climate-related health effects among elected or appointed officials, business leaders, and leaders of the healthcare delivery system. This uncertainty signals a lack of necessary dialogue and focus on climate change among key Michigan stakeholders. This is consistent with Kalafatis and Lemos (2017) identifying a lack of climate entrepreneur leaders, that is, few public officials clearly associated with pushing for climate action, in the Great Lakes region. A lack of knowledge among key leaders may result in a lack of priority and resources for preparedness.

Our survey also examined the extent to which LHD officials look to State and federal agencies for expertise. One third and one half of respondents perceived the MDHHS (32%) and the CDC (53%), respectively, as having *a lot* or *a good deal* of expertise in creating effective adaptation plans. The median response for MDHHS was *some* expertise (3 on a scale of 1 *none* to 5 *a lot* of expertise) and *a good deal* of expertise for CDC (4 out of 5). No respondents identified the state as having *a lot* of expertise. Importantly, over a quarter reported they did not know either MDHHS or CDC’s level of expertise. Because LHDs rely on state and national agencies to inform their adaptation plans and actions, local capacity could be further challenged by doubt or uncertainty regarding the level of expertise available from these institutions (Roser-Renouf et al. 2016). Additionally, it is difficult for the MDHHS and CDC to provide their climate expertise to LHDs whose leadership is not emphasizing the impact of climate change.

### Research Question 3: Perceived value of State and Centers for Disease Control and Prevention frameworks

Efforts by the MDHHS and CDC to guide LHDs as they prepare for the health impacts of climate change did not appear to have gained much traction in Michigan in the past decade (Michigan Department of Community Health 2010). Notably, Michigan faces challenges with trust between different levels of government (Boufides et al. 2019). This survey found relatively low levels of perceived familiarity and utility of both the MICHAP strategic plan and the CDC BRACE framework. Beyond asking LHD respondents to identify key health effects related to climate change, the survey did not otherwise explore respondents’ familiarity with the concepts in these documents even if they did not recognize the names of the strategies and frameworks.

Importantly, the lack of familiarity among LHDs with resources available to them from other levels of government is a missed opportunity on two levels. First, the existence of state and national programs signals the importance of this topic to LHD senior managers and stakeholders and could be used to educate decision makers and the public regarding the connection between climate and public health. Second, state and federal frameworks can fill the gap of limited local knowledge and capacity, and support local districts’ preparedness for climate change. However, the data, analyses, tools, and experience are not being utilized to bridge the gap identified; namely that LHDs know that climate change influences health and yet jurisdictions are not prioritizing or acting on this knowledge.

Recognizing the need for additional action to protect health, in September 2020, the Governor of Michigan established state goals for decarbonization by 2050 and issued the Michigan Healthy Climate Plan, building from the BRACE framework and MICHAP strategy (Cameron and Ferguson 2016). The Governor directed the Michigan Department of Environment, Great Lakes, and Energy (EGLE) to implement the plan and established a Council on Climate Solutions with a range of stakeholders from multiple sectors to advise the statewide process. In the forthcoming MICHAP update, MDHHS can work to ensure that LHDs recognize the health impacts from climate change, recommend that solutions consider public health and equity impacts, and that frontline communities benefit from new resources and projects. MDHHS can also educate other departments and the broader public health community to leverage established relationships with LHDs to promote the climate and health messaging and frame the solutions in ways that align with existing processes, incentives, and requirements.

### National context

The challenge to state and federal agencies is to reach LHDs who are currently under-funded and marginally staffed to satisfy standard programmatic demands along with emergent pandemic responses. States are increasingly important agents in US climate efforts, often through gathering and interpreting data (e.g., complex climate modeling, systematic reviews of scientific literature), creating policies and programs that promote or inhibit adaptation at other scales of governance, applying influence or political pressure on local and federal stakeholders, and by serving as test sites for climate innovation (Stults and Woodruff 2017). Although many of these actions are not specifically designed to address the health effects of climate change (Angel et al. 2018), they often shape the conditions that promote or hinder population health locally.

Increasingly, climate justice advocates are calling for structural changes to mitigate greenhouse gases, evolve community assets to be more resilient to climate stresses, and build communities that routinely promote health for all people (Perkins 2019; Schlosberg 2012). Health equity is especially important in low-wealth communities in which overall risk is significantly increased by the greater frequency and severity of climate-related hazards due to high population exposure and limited adaptive capacity. In Michigan, for example, recurrent flooding in combination with other physical (e.g., heat, air pollution) and social exposures (e.g., racist housing segregation) exacerbated by climate change and current infrastructure erodes local coping capacity. This erosion results in household and neighborhood deficits in long-term adaptive capacity and increases cumulative health risks (Gregg et al. 2012; Detroiters Working for Environmental Justice 2017; City of Marquette 2013).

An effective approach to necessary climate preparations will require local government, business, and political leaders to all have the knowledge and capacity to actively participate. More community resilience is necessary at the same time that local public health resources are under more pressure than ever. The ability of LHDs to respond to and plan for climate-related health risk is predicted by both their risk perceptions and their funding levels (Roser-Renouf et al. 2016). In light of the COVID-19 pandemic and economic downturn, the capacity of LHDs to plan for and respond to climate change may become further constrained. However, this planning is increasingly vital as many state and local adaptation plans rely on transporting and gathering people to shelter them from storms, cool them during heat waves, or treat them for climate-related health effects in health care systems simultaneously contending with the impact of an infectious disease pandemic. Thus, knowledge of local perceptions is essential to researchers, practitioners, and others as they seek to effectively help communities prepare for and adapt to climate change. (Schramm et al. 2020).

While action at global, national, regional, and local scales is needed, climate-related health impacts are most pronounced at the local level. In previous national surveys, most LHD directors agreed that climate change is already happening locally and would cause local health problems within 20 years, yet few agreed that their department had expertise or resources to address the issue (Maibach et al. 2008; Roser-Renouf et al. 2016). These findings have been replicated in other areas such as California, New York, and Oregon (Bedsworth 2009; Carr et al. 2012; Vynne and Doppelt 2009; Syal et al. 2011).

This perspective assumes that climate change will not necessitate substantial changes to public health practice beyond gradually increased resources and program expansion (Hess et al. 2012). Yet, US local governments have unique authorities and tools to address climate impacts including data gathering, monitoring and surveillance, capacity-building, financing, policy making or regulation, advocacy, education, and risk management. The view that public health’s readiness is contingent on sufficient support places the emphasis on bolstering rather than reconfiguring public health practice and systems. To the extent that transformational approaches are needed to protect public health from a changing climate, the catalytic power of state and federal agencies will become increasingly important. Although there is no national requirement for climate health planning in the USA, the BRACE framework is an important first step to help states and communities to consider climate change, establish common data sets, such as climate projections, to be used for planning, and develop appropriate public health mitigation, adaptation and resilience options, including incremental and transformative strategies.

### Strengths

Surveys of Michigan LHD perceptions on climate change and health are lacking, and this study contributes important data regarding Michigan local public health officials’ perceptions of climate change and its associated health impacts. Our partnership with MALPH likely boosted our response rate because the survey was conveyed by a trusted source.

The survey utilized a previously tested and well-established instrument for question text and format (Maibach et al. 2008). The ability to respond anonymously decreases virtuous response bias and has the potential to more accurately reflect views than other techniques such as key informant interviews. This survey also expanded on two national surveys conducted by Maibach et al. 2008 and Roser-Renouf et al. 2016 to explore perceptions regarding the MICHAP strategic plan and the CDC’s BRACE framework.

### Limitations

Our non-random Michigan respondents may differ from the nationally representative sample of previous surveys, limiting the comparisons that could be made. In addition, the effects of climate change (e.g., wildland fires in Western U.S., stronger storms in ocean coastal communities) may be less observable in midwestern states like Michigan than in other states included in a nationally representative sample, rendering it less likely that respondents would identify and affirmatively acknowledge them (Brown et al. 2012). Additionally, the sample size of the current study (*n* = 34) was small, limiting the generalizability of these results or our ability to assess subset distinctions such as attitudes in rural districts compared to other areas.

The 2019 survey was distributed to a small, non-randomized and non-representative sample. This study did not use randomized sampling techniques; samples were instead drawn from a trusted professional association of LHD officials with the intent of snowball sampling (respondents passing the survey to colleagues). Thus, the results of this study may be affected by selection bias because those with higher interest or stronger views on the topic may have been more motivated to respond to the survey. Additionally, the study had limited follow-up and did not offer incentives for survey completion.

The survey was not designed to explore underlying rationales for respondent perceptions and there are difficulties in separating nuanced responses. For example, Michigan may be perceived to be experiencing fewer extreme climate events relative to other states (e.g., western wildland fires, southwestern droughts, southern heat, or hurricane-prone coastal communities). Some Michigan responses about climate impacts may reflect a high level of nuanced knowledge about the impacts and modeling, and the expectation that Michigan will be less affected than other areas in the near term based on current modeling. Our novel survey questions were at the end of the instrument and received fewer answers (*n* < 15) compared to the earlier questions (*n* = 34).

## Conclusion

This research revealed patterns in the perceptions of climate-related health effects among Michigan local public health officials that are broadly similar to those reported nationwide. Approximately 60% of Michigan LHD officials indicated that their jurisdictions have already experienced climate change, and 76% indicated that their jurisdictions will experience climate change in the next two decades (*n* = 34). However, many fewer respondents (35%) indicated that dealing with the health impacts of climate change was a priority in their departments. Despite increasing emphasis within the scientific community about the connection between climate change and public health, the survey does not indicate perceptions of priority or urgency in addressing climate determinants of health among Michigan LHDs.

A substantial number of respondents indicated that they did not know whether specific health issues in their jurisdictions had been or would be exacerbated by climate change in the future. Many respondents similarly indicated that they did not know the level of expertise of state and federal agencies in creating health-focused adaptation plans or the level of knowledge possessed by community stakeholders.

The survey also identified a perceived deficit of individual and organizational familiarity and utility of both the MICHAP strategic plan and the BRACE framework. This finding indicates that LHD officials experience a lack of institutional support to contend with the health impacts of climate change, and that additional efforts are needed to familiarize local officials with the resources available to them. While the 2019 Michigan survey has limitations that should be taken into consideration, including a small sample size and the potential for selection bias, this research can be utilized to better understand the state of climate change perceptions in Michigan’s LHDs with implications for other areas.

New resources are beginning to flow to LHDs for climate change adaptation (e.g., the American Public Health Association (APHA) guidebook (Rudolph et al. 2018), CDC cross-sector collaboration toolkit (Centers for Disease Control and Prevention 2020)). Additionally, the CDC Foundation recommends best practices including the presence of climate action committees and partnerships that include local public health officials and the development of locally relevant climate and health messaging (CDC Foundation 2020). Both of these practices, especially if adopted together, may work to bridge the gaps identified in this study between knowledge and explicit action in the state of Michigan. In most states, however, LHDs have specific powers and duties under the Public Health Code which they must fulfill in order to retain their state accreditation, and funding is often directly tied to the essential services, either through fees (e.g., sanitation inspections of restaurants or campgrounds) or grants for a specific duty (e.g., immunization clinics). A key challenge is for public health agencies to address climate determinants of health in a way that fit into the LHDs’ traditional roles.

Recognizing these challenges, state and federal coordination with LHDs needs to be more explicitly included and funded in both state and federal climate action plans and budgets. One approach being explored in Michigan and elsewhere is incorporating climate impacts into the Community Health Needs Assessments that are required for accreditation. In addition to community needs assessments, other tools to advance climate objectives include emergency preparedness planning, community resources and infrastructure, health education programs, training, and development and use of data, surveillance, and tracking. It is important to increase support and engage with the organizations that are already providing health and human services to community populations and could become partners in addressing the climate-related impacts. Future LHD surveys based on the Maibach et al. (2008) model may indicate to what extent these resources and efforts are making an impact and what other initiatives can contribute to positive climate determinants of health.

## Supplementary Information

ESM 1 (PDF 128 kb)
