# Supplementary material for: Assessing perceptions and priorities for health impacts of climate change within local Michigan health departments
Source: J Environ Stud Sci. 2021 May 11;11(4):595–609. doi: 10.1007/s13412-021-00679-0 (PMC8112836; doi:10.1007/s13412-021-00679-0)
Supplement: Supplementary file 1 — (PDF 128 kb) [file 13412_2021_679_MOESM1_ESM.pdf]

Supplementary Materials

Survey Instrument

Thank you for completing this survey. The purpose of this study is to explore the perceptions of climate change and its anticipated impact on human health within county health departments in Michigan. Please answer the following questions as completely and honestly as possible.

1. What is your position/job title? ______________________________

2. What county/district is served by your health department: ________________________

3. Approximately, how long have you worked in your current department:
   a. Less than one year
   b. 1 to 5 years
   c. 5 to 10 years
   d. 10 to 15 years
   e. More than 15 years

4. Please indicate how strongly you agree or disagree with the following statements.

1 = Strongly Disagree	2 = Disagree	3 = Don't Know	4 = Agree	5 = Strongly Agree

1	2	3	4	5	My jurisdiction has experienced climate change in the past 20 years.
1	2	3	4	5	My jurisdiction will experience climate change in the next 20 years.
1	2	3	4	5	Preparing to deal with the public health effects of climate change is a priority for my health department.

5. Has climate change already affected the following health issues in your jurisdiction?:

1 = Yes	2 = No	D = Don't Know

1	2	3	D	Heat waves and heat-related illness
1	2	3	D	Waterborne disease
1	2	3	D	Vector-borne disease
1	2	3	D	Air quality and respiratory diseases
1	2	3	D	Unsafe/ineffective sewage and septic operation
1	2	3	D	Health care services for people with chronic conditions during service disruptions, such as extreme weather events
1	2	3	D	Anxiety, depression, or other mental health conditions
1	2	3	D	Injury from extreme weather events

6. Over the next 20 years, will climate change make this issue *more* common or severe, *less* common or severe, or will it remain the *same* in your jurisdiction?:

1 = Less Common/Severe	2 = Same	3 = More Common/Severe	D = Don't Know

1	2	3	D	Heat waves and heat-related illness
1	2	3	D	Waterborne disease
1	2	3	D	Vector-borne disease
1	2	3	D	Air quality and respiratory diseases
1	2	3	D	Unsafe/ineffective sewage and septic operation

1 2 3 D	Health care services for people with chronic conditions during service disruptions, such as extreme weather events
1 2 3 D	Anxiety, depression, or other mental health conditions
1 2 3 D	Injury from extreme weather events

7. How much do the following stakeholders in your county/district know about the potential health impacts of climate change?

1 = None	2 = A Little	3 = Some	4 = A Good Deal	5 = A Lot	D = Don't Know

1 2 3 4 5 D	You/yourself personally
1 2 3 4 5 D	Relevant senior managers in my health department
1 2 3 4 5 D	Relevant appointed officials- such as environmental, agricultural, wildlife, energy, and transportation officials
1 2 3 4 5 D	Relevant elected officials
1 2 3 4 5 D	Business owners
1 2 3 4 5 D	Leaders of the health care delivery system- including hospitals and medical groups

8. How much expertise do you feel the following groups have in creating effective climate change adaptation plans focused on public and community health?

1 = None	2 = A Little	3 = Some	4 = A Good Deal	5 = A lot	D = Don't Know

1 2 3 4 5 D	Your local health department
1 2 3 4 5 D	Michigan Department of Health and Human Services (MDHHS)
1 2 3 4 5 D	The Centers for Disease Control and Prevention (CDC)

9. How familiar are the following individuals/groups with the CDC's BRACE (Building Resilience Against Climate Effects) framework?:

Not familiar	2 = Slightly familiar	3 = Moderately familiar	4= Considerably familiar	5 = Highly familiar	D = Don't Know

1 2 3 4 5 D	Myself personally
1 2 3 4 5 D	My health department
1 2 3 4 5 D	Relevant appointed officials- such as environmental, agricultural, wildlife, energy, and transportation officials
1 2 3 4 5 D	Relevant elected officials
1 2 3 4 5 D	Michigan Department of Health and Human Services

10. To what degree has the CDC BRACE framework been a valuable resource for your department?:
    a. Not valuable
    b. Slightly valuable
    c. Moderately valuable
    d. Considerably valuable
    e. Highly valuable
    f. Don't know

11. How familiar are the following individuals/groups with the MDHHS MICHAP (Michigan Climate and Health Adaptation Plan)?:

Not familiar 2 = Slightly familiar 3 = Moderately familiar 4= Considerably familiar 5 = Highly familiar DK = Don't Know

1	2	3	4	5	Myself personally
1	2	3	4	5	My health department
1	2	3	4	5	Relevant appointed officials- such as environmental, agricultural, wildlife, energy, and transportation officials
1	2	3	4	5	Relevant elected officials

12. To what degree has MICHAP been a valuable resource for your department?:
    a. Not valuable
    b. Slightly valuable
    c. Moderately valuable
    d. Considerably valuable
    e. Highly valuable
    f. Don't know
